# ﻿Increasing knowledge of *Cymbiapophysa* Gabriel & Sherwood, 2020 (Araneae, Theraphosidae): general distribution, key to species, and three new species from Ecuador

**DOI:** 10.3897/zookeys.1178.105703

**Published:** 2023-09-01

**Authors:** Pedro Peñaherrera-R.

**Affiliations:** 1 Universidad San Francisco de Quito USFQ, Colegio de Ciencias Biológicas y Ambientales, Instituto de Biodiversidad Tropical IBIOTROP, Laboratorio de Zoología Terrestre, Museo de Zoología, Quito 170901, Ecuador Universidad San Francisco de Quito USFQ Quito Ecuador

**Keywords:** Andes, Azuay, biogeography, Cotopaxi, distribution, Pichincha, tarantula, taxonomy

## Abstract

Three new species of *Cymbiapophysa* Gabriel & Sherwood, 2020 are described from south, central, and north-western Ecuador, showing the wide range of distribution that this genus has in Ecuador and its biogeographical provinces. These three new species are easily differentiated from other congeners based on keel morphology of the male palpal bulb. Supplementary information about the locality of *C.magna* Sherwood, Gabriel, Brescovit & Lucas, 2021 is provided, alongside additional data on morphology and some commentaries about the general distribution and biogeography of *Cymbiapophysa*. Additionally, a taxonomic key for males of *Cymbiapophysa* species is presented, based on the palpal bulb morphology.

## ﻿Introduction

*Cymbiapophysa* Gabriel & Sherwood, 2020 is a recently described genus that includes small to medium sized theraphosid spiders diagnosed from most other members of Theraphosidae Thorell, 1869 by the combination of: a distal-retrolateral apophysis on the male cymbium, an embolic ridge, a prolateral ridge, a ventral median depression in the male palpal bulb, and a twin spermathecae with short, squat receptacles with a rectangular guard plate (Gabriel and Sherwood 2020; Sherwood et al. 2021a). This neotropical group currently has four valid species distributed in Colombia, Ecuador, and possibly Peru (Gabriel and Sherwood 2020; Sherwood et al. 2021a; WSC 2023). For Ecuador, the only known species of *Cymbiapophysa* are *C.velox* (Pocock, 1903) and *C.seldeni* Sherwood & Gabriel, 2023, both described from specimens collected by W. F. H. Rosemberg at Paramba and Pambelar in the case of *C.velox*, and Carondelet for *C.seldeni*, in the Province of Esmeraldas, northwestern Ecuador (Pocock 1903; Gabriel and Sherwood 2020; Sherwood and Gabriel 2023).

During the revision of specimens of Mygalomorphae deposited at the Museo de Zoología of Universidad San Francisco de Quito and Instituto Nacional de Biodiversidad del Ecuador, three new species of *Cymbiapophysa* were identified. This paper aims to describe these three new species based on their distinctive male palpal bulb morphology, alongside some comments on maxillae, coxae, trochanters, tibiae and metatarsi, the possible locality *C.magna* Sherwood, Gabriel, Brescovit & Lucas, 2021, and discussion on the general distribution and biogeography of *Cymbiapophysa* species. A taxonomic key for males of *Cymbiapophysa* is presented.

## ﻿Materials and methods

Specimens were examined and measured under an Olympus SZX16 stereomicroscope with an Olympus DP73 digital camera and an Olympus CX22 microscope with an OMAX A35180U3 digital camera. Measurements were recorded with Micro Imaging Software CellSens for Olympus. All measurements are presented in millimetres. Compound images were obtained by stacking a series of photographs taken at different depths using an Olympus DP73 digital camera and then processed with the staking software of Photoshop and editing tools. Examined specimens are deposited at Instituto Nacional de Biodiversidad, Ecuador (**INABIO**) and Museo de Zoología, Universidad San Francisco de Quito, Ecuador (**ZSFQ**).

Biogeographic classification follows the proposal by Morrone (2014), with modifications proposed by Cisneros-Heredia and Yánez-Muñoz (2007) and Cisneros-Heredia (2006, 2007, 2019). Ecuadorian classification of ecosystems follows MAE (2013). General description and measurements follow standards proposed by Gabriel and Sherwood (2020) and Sherwood et al. (2021a) for the genus *Cymbiapophysa*. Leg spination description follows definitions and terminology proposed by Petrunkevitch (1925) and Bertani (2001). Palpal bulb terminology follows Bertani (2000), Gabriel (2016), Gabriel and Sherwood (2020), Sherwood et al. (2021a), and Ferretti et al. (2023). Subtypes of urticating setae follow Kaderka et al. (2019). The key to species of *Cymbiapophysa* is based on available information from Perafán and Valencia-Cuéllar (2018), Gabriel and Sherwood (2020), Sherwood et al. (2021a), and Sherwood and Gabriel (2023).

The type localities of the previously known species of *Cymbiapophysa*, excluding *C.yimana*, were obtained from the original descriptions of Pocock (1903), Perafán and Valencia-Cuéllar (2018), Sherwood et al. (2021a), Sherwood and Gabriel (2023). Additional information about *C.velox* localities were obtained from Peters (1955), Brown (1941), and Lynch and Duellman (1997). Map was made using ArcMap.

Morphological abbreviations: Somatic: **AME**, anterior median eyes; **ALE**, anterior lateral eyes; **PME**, posterior median eyes; **PLE**, posterior lateral eyes. Male genitalia: **A**, apical keel; **D**, dorsal median depression; **PI**, prolateral inferior keel; **PS**, prolateral superior keel; **PACK**, prolateral accessory central keel; **PAIK**, prolateral accessory inferior keel; **RI**, retrolateral inferior keel; **RS**, retrolateral superior keel; **ER**, embolic ridge; **PC**, prolateral crease; **PR**, prolateral ridge; **PAR**, prolateral apical ridge; **TH**, tegular heel.

## ﻿Results

### ﻿Taxonomy

#### ﻿ Cymbiapophysa falconi sp. nov.

Taxon classificationAnimaliaAraneaeTheraphosidae

6C333A59-F295-524F-A927-984B69CBFD0E

https://zoobank.org/FFA3DF14-2900-49EC-BC81-E8FE1A152258

Figs 1 , 2 , 3 , 6A , 7A , 8 , 9C, D , 10

##### Material examined.

***Holotype***: Republic of Ecuador • 1 ♂; province of Azuay, canton La Unión, Parish of Chordeleg, Valley of Yunguilla, near the Chantaco river; -3.2421, -79.2685, 1620 m a.s.l.; 03 November 2019; J. Falcón-Reibán & A.Velez leg.; AE-0005. ***Paratype***: Republic of Ecuador • 1 ♂, same data as holotype; 03 November 2019; J. Falcón-Reibán and A. Velez leg.; AE-0004.

##### Diagnosis.

*Cymbiapophysafalconi* sp. nov. can be distinguished from other species by the morphology of the male palpal bulb. This new species can be differentiated from *C.homeroi* sp. nov. by the presence of a disjunct and distally slightly serrated PACK keel, PS keel longer than PI keel, and D weakly developed (continuous and slightly serrated PACK keel, PS keel as long as PI keel, and D more developed in *C.homeroi* sp. nov.); from *C.carmencita* sp. nov. by the presence of a disjunct and distally slightly serrated PACK keel, smooth PI keel, continuous and weakly developed A keel, PS keel longer than PI keel, and absence of Type III urticating setae (continuous and slightly serrated PACK keel, slightly serrated PI keel, disjunct and strongly developed A keel, PS as long as PI keel, and presence of Type III urticating setae in *C.carmencita* sp. nov.); from *C.velox* and *C.yimana* by the presence of a disjunct and distally slightly serrated PACK keel, PS keel longer than PI keel, and absence of Type III urticating setae (PACK keel(s) absent, PS keel as long as PI keel, and presence of Type III urticating setae in *C.velox* and *C.yimana* (Gabriel and Sherwood 2020; Sherwood et al. 2021a)); from *C.marimbai*, *C.magna*, and *C.seldeni* by the presence of a disjunct and distally slightly serrated PACK keel, absence of a tibial apophysis, D weakly developed, and absence of Type III urticating setae (two PACK keels present, tibial apophysis present, D well-developed, and presence of Type III urticating setae in *C.marimbai*; PACK keel (s) absent, tibial apophysis present, D developed, and presence of Type III urticating setae in *C.magna*; PACK keel (s) absent, tibial apophysis present, and D weakly-developed in *C.seldeni* (Perafán and Valencia-Cuéllar 2018; Sherwood et al. 2021a; Sherwood and Gabriel 2023). *Cymbiapophysafalconi* sp. nov. can further be distinguished from all other species by the presence of a well-developed RI keel projected to the prolateral face (straight and weakly developed RI keel in *C.homeroi* sp. nov., *C.velox*, and *C.yimana*; RI keel absent in *C.carmencita* sp. nov., *C.magna*, *C.marimbai*, and *C.seldeni* (Perafán and Valencia-Cuéllar 2018; Gabriel and Sherwood 2020; Sherwood et al. 2021a; Sherwood and Gabriel 2023)).

##### Description.

**Figure 1.**
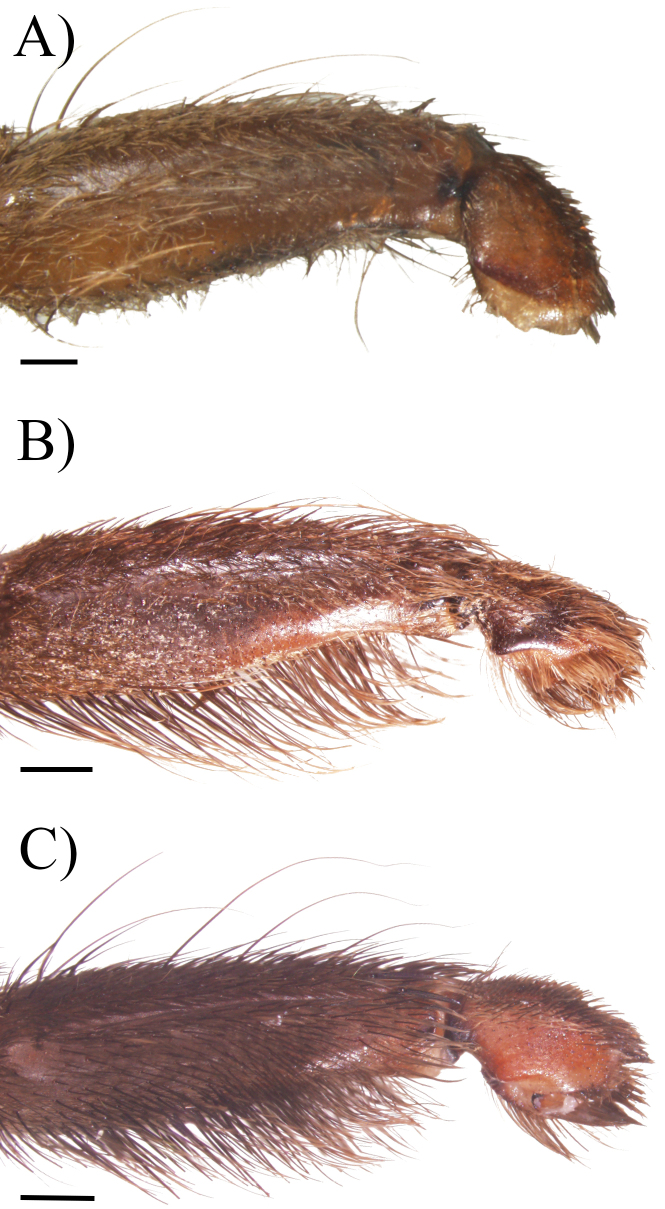
Leg I, metatarsus morphology, retrolateral view **A***Cymbiapophysafalconi* sp. nov. male holotype (AE-0005) **B***Cymbiapophysahomeroi* sp. nov. male holotype (ZSFQ-i11577) **C***Cymbiapophysacarmencita* sp. nov. male holotype (ZSFQ-i11578). Scale bars: 0.5 mm (**A**); 1 mm (**B, C**).

**Figure 2.**
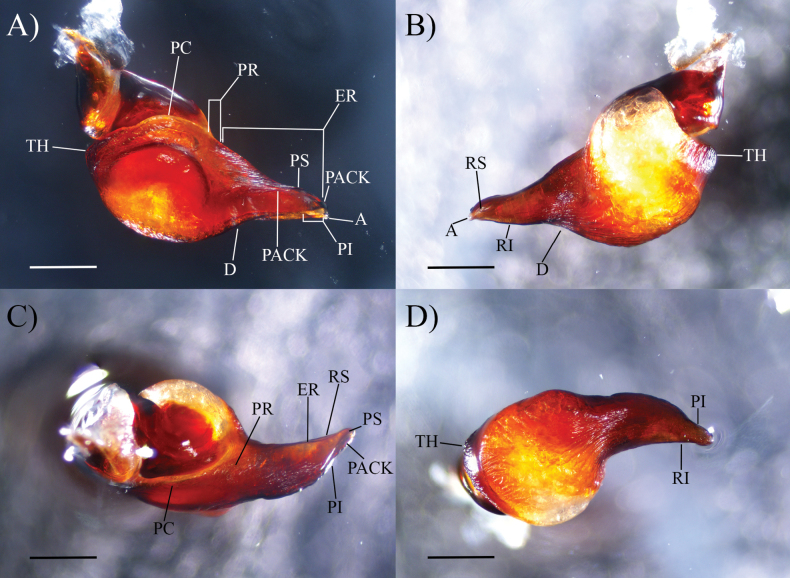
*Cymbiapophysafalconi* sp. nov. male holotype (AE-0005), palpal bulb (left hand side): **A** prolateral view **B** retrolateral view **C** dorsal view **D** ventral view. Abbreviations: A, apical keel; D, dorsal median depression; PI, prolateral inferior keel; PS, prolateral superior keel; PACK, prolateral accessory central keel; PAIK, prolateral accessory inferior keel; RI, retrolateral inferior keel; RS, retrolateral superior keel; ER, embolic ridge; PC, prolateral crease; PR, prolateral ridge; TH, tegular heel. Scale bars: 0.5 mm.

***Male holotype*** (AE-0005): Total length including chelicerae: 19.74. Carapace: length 9.11, width 7.74. Caput: not raised. Ocular tubercle: slightly raised, length 0.98, width 1.46. Eyes: ALE > AME, PLE > AME, PLE > PME, anterior eye row straight, posterior row slightly recurved. Clypeus: narrow; clypeal fringe short. Fovea: straight. Chelicera: length 2.52, width 1.52. Abdomen: length 8.11, width 4.66. Maxilla with 130–183 cuspules covering approximately 50% of the proximal edge. Labium: length 1.25, width 1.49, with 66 cuspules most separated by 0.5–1.0× the width of a cuspule. Labio-sternal mounds joined along the entire base of the labium. Sternum: length 4.18, width 3.74, with three pairs of sigilla. Tarsi I–IV fully scopulate, tarsi I–III divided by narrow strip of longer and thicker setae, Tarsus IV divided by wide strip of longer and wider setae. Metatarsal scopulae: I 50%; II 60%; III 30%; IV 10%. Lengths of legs and palpal segments: see Table 1, legs IV, I, II, III. Spination: Leg I: femur p 0-0-1; tibia p 0-1-1-0, r 0, v 0-1-1-0(3ap); metatarsus v 1-0-0(3ap). Leg II: femur p 0-0-1; tibia p 0-1-1-0, v 0-1-0(3ap); metatarsus p 0-0-1, r 1-0-0(3ap).Leg III: femur p 0-0-1, r 0-0-1; patella r 0-0-1, v 0; tibia p 1-1-1, r 1-1-1, v 0-2-0(3ap); metatarsus p 1-1-1, r 1-0-1, v 0-1-1-1-1-0(6ap). Leg IV: femur d 0-0-1; patella r 0-1-0; tibia p 1-0-1, r 1-1-1, v 1-1-1(3ap); metatarsus p 1-1-1-1, r 1-1-1, v 1-1-2-1-1-1-1 (7ap). Pedipalp: femur p 0-0-1; tibia p 1-1-2-1-2(ap). Palpal cymbium with rounded weakly developed retrolateral apophysis (Fig. 1A). Leg I lacking tibial apophyses. Femur III laterally incrassate. Palpal tibia slightly laterally incrassate. Metatarsus I straight. Posterior lateral spinnerets with three segments, basal 1.70, median 0.57, digitiform apical 1.44. Posterior median spinnerets with one segment. Palpal bulb (Fig. 2) with weakly-developed and rounded TH. PS, PACK, and RI keels well-developed; PI, RS and A keels weakly developed. PS keel longer than PI keel, PS extending for one third of the length of the embolus. PACK keel extending to half the length of the embolus, disjunct with the distal part slightly serrated. RI keel extending to half the length of the embolus and distally projected to the prolateral face. ER disjunct from PC forming a PR, PAR absent. PC narrow and constricted in posterior half. D weakly developed. Ventral and dorsal faces with a rugulose area. Only Type Ia urticating setae present, Type III urticating setae absent. Colour (after four years in preservative): generally with a brown colouration and setae with a pale grey colour.

**Table 1.** *Cymbiapophysafalconi* sp. nov. male holotype (AE-0005), podomere measurements.

	Femur	Patella	Tibia	Metatarsus	Tarsus	Total
**I**	7.75	4.09	7.93	5.74	4.57	30.08
**II**	7.74	3.42	6.86	5.92	4.37	28.31
**III**	7.02	3.77	6.74	5.57	4.5	27.60
**IV**	6.23	3.8	7.82	10.69	4.9	33.44
**Palp**	5.16	2.81	4.25	-	1.23	13.45

##### Variation.

The paratype male (AE0004) has carapace length 7.28, width 7.25, abdomen length 5.56, width 8.27, maxilla with 111–170 cuspules, labium with 56 cuspules. It presents the same palpal bulb morphology, except for a more elongated PACK keel, extending almost more than half of the PS keel.

##### Remarks.

The male holotype (AE-0005) and paratype (AE-0004) of *Cymbiapophysafalconi* sp. nov. are deposited in the invertebrate collection of Instituto Nacional de Biodiversidad del Ecuador. However, the Mygalomorphae collection of this institution is not yet properly managed or digitised. Therefore, these specimens do not have the current coding numeration (INABIO-MECN-AR) like the rest of invertebrates of this collection, but each specimen presents a unique catalogue specimen code, which is used here.

##### Etymology.

The specific epithet is an eponym for José M. Falcón-Reibán, a great friend and colleague who introduced me to the curiosity of studying tarantulas, by showing me an unknown tarantula back in 2020.

##### Distribution.

*Cymbiapophysafalconi* sp. nov. is only known from its type locality, in the Valley of Yunguilla, near the Chantaco river, at 1620 m, Province of Azuay, on the southwestern slopes of the Cordillera Occidental of the Andes of Ecuador.

##### Ecology.

**Figure 3.**
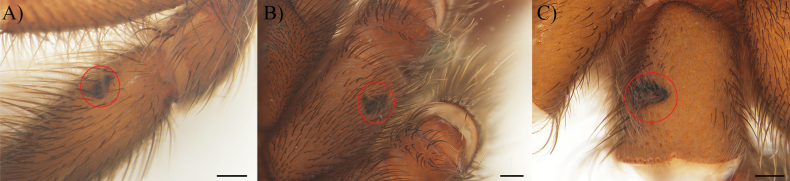
Scarification on leg segments in *Cymbiapophysafalconi* sp. nov. paratype (AE-0004): **A** tibia IV (right hand side), ventral view **B** coxa II (right hand side), ventral view. Holotype (AE-0005): **C** coxa III (left hand side), ventral view. Scale bars: 0.5 mm.

*Cymbiapophysafalconi* sp. nov. inhabits semi-deciduous shrubland in the southern inter-Andean valleys, in the Western Ecuador biogeographic Province (Fig. 10). *Cymbiapophysafalconi* sp. nov. is considerably rare to find; except during the wet season between February and May where males and females (uncollected female specimens) are easy to find below small shrubs and cactus (José Falcón-Reibán, pers. Comm.). Both known males show small and irregular scarifications in coxa II–III and femur IV (Fig. 3). The origin of these scarifications is unknown but could have been produced by preys trying to defend themselves, as observed in other small theraphosids predating large beetles in captivity (Peñaherrera-R. pers. Obs.).

#### ﻿ Cymbiapophysa homeroi sp. nov.

Taxon classificationAnimaliaAraneaeTheraphosidae

18C560DF-F8E1-57DC-A32C-32EADCE98A6C

https://zoobank.org/B36F5C8C-9E34-4741-B70D-178D248FF378

Figs 1 , 4 , 10

##### Examined material.

***Holotype***: Republic of Ecuador • 1 ♂; Province of Santo Domingo de los Tsachilas, Canton Santo Domingo, Parish of San José de Alluriquín, Reserve Rio Guajalito, trail Los Españoles; -0.2338, -78.7939, 2260 m a.s.l.; 26 November 2015; M. Costales leg.; ZSFQ-i11577.

##### Diagnosis.

*Cymbiapophysahomeroi* sp. nov. can be distinguished from other species by the morphology of the male palpal bulb from *C.falconi* sp. nov. by the presence of a continuous and slightly serrated PACK keel, PS keel as long as PI keel, and D developed (disjunct and distally slightly serrated PACK keel, PS keel longer than PI keel, and D weakly developed in *C.falconi* sp. nov.); from *C.carmencita* sp. nov. by the presence of a slightly serrated PACK keel and smooth PI keel, developed RS keel, weakly developed RI keel, weakly developed A keel, D developed, and absence of PAIK keel and Type III urticating setae (slightly serrated PACK and PI keels, absence of RS and RI keels, disjunct and developed A keel, developed PAIK keel, D weakly developed, and presence of Type III urticating setae in *C.carmencita* sp. nov.); from *C.velox* and *C.yimana* by palpal bulb morphology with the presence of a continuous and slightly serrated PACK keel, D developed, and absence of Type III urticating setae (PACK keel(s) absent, D weakly developed, and presence of Type III urticating setae in *C.velox* and *C.yimana* (Gabriel and Sherwood 2020; Sherwood et al. 2021a)); from *C.marimbai*, *C.magna*, and *C.seldeni* by the presence of a single slightly PACK keel and a RI keel, having the PS keel as long as PI keel, absence of a tibial apophysis, D developed, and absence of Type III urticating setae (two PACK keels present, RI keel absent, PS keel longer than PI, tibial apophysis present, D well-developed, and presence of Type III urticating setae in *C.marimbai*; PACK and RI keels absent, PS keel longer than PI, tibial apophysis present, D developed, and presence of Type III urticating setae in *C.magna*; PACK keel (s) absent, PS keel as long as PI keel, tibial apophysis present, and D weakly-developed in *C.seldeni* (Perafán and Valencia-Cuéllar 2018; Sherwood et al. 2021a; Sherwood and Gabriel 2023)). Additionally, *Cymbiapophysahomeroi* sp. nov. can further be distinguished from all other species, by the exception of *C.carmencita* sp. nov., by the presence of a well-developed PI keel (developed PI keel in *C.magna*, *C.marimbai*, *C.seldeni*, and *C.yimana*; weakly developed RI keel in *C.falconi* sp. nov. and *C.velox* (Perafán and Valencia-Cuéllar 2018; Gabriel and Sherwood 2020; Sherwood et al. 2021a; Sherwood and Gabriel 2023)).

##### Description.

**Figure 4.**
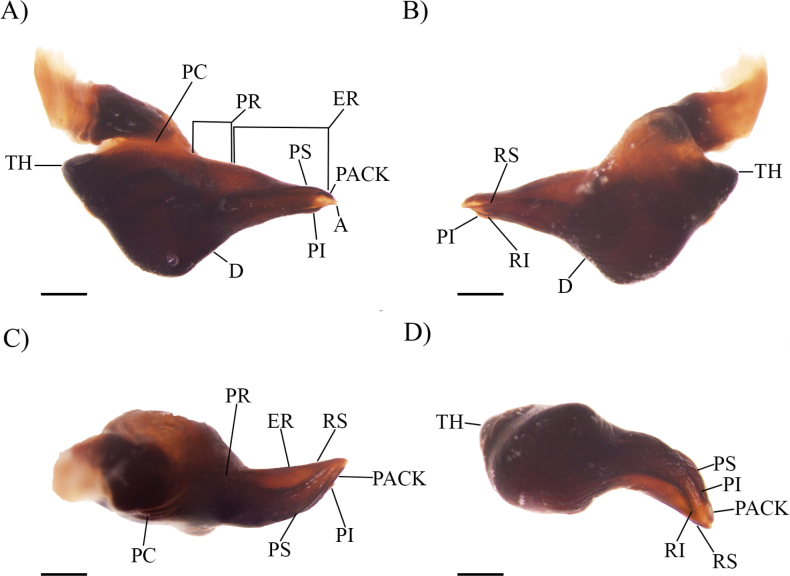
*Cymbiapophysahomeroi* sp. nov. male holotype (ZSFQ-i11577), palpal bulb (left hand side): **A** prolateral view **B** retrolateral view **C** dorsal view **D** ventral view. Scale bars: 0.5 mm.

***Male holotype*** (ZSFQ-i11577): Total length including chelicerae: 24.75. Carapace: length 12.95, width 11.10. Caput: slightly raised. Ocular tubercle: raised, length 1.14, width 2.05. Eyes: AME > ALE, AME > PLE, PLE > PME, anterior eye row straight, posterior row slightly recurved. Clypeus: narrow; clypeal fringe short. Fovea: straight. Chelicera: length 2.14, width 2.23. Abdomen: length 9.66, width 5.76. Maxilla with 112–146 cuspules covering approximately 60% of the proximal edge. Labium: length 1.80, width 2.00, with 52 cuspules most separated by 0.5–1.0× the width of a cuspule. Labio-sternal mounds joined along the entire base of the labium. Sternum: length 4.87, width 4.89, with three pairs of sigilla. Scopulation, leg measurements and spination unknown due to the fragile state of the specimen. Palpal cymbium with conical well-developed retrolateral apophysis (Fig. 1B). Leg I lacking tibial apophyses. Femur III and palpal tibia laterally incrassate. Metatarsus I straight. Palpal bulb (Fig. 4) with developed and triangular TH. PS, PACK, and PI keels well-developed; RS keel developed; RI and A keels weakly developed. PS as long as PI keel. Small and slightly serrated PACK keel extending less than a half the length of the embolus. PI visible in retrolateral and prolateral views. ER disjunct from PC forming a PR, PAR absent. PC narrow and slightly constricted in posterior half. D developed. Ventral face with rugulose area. Type Ia urticating setae present, Type III urticating setae absent. Colour: after thirteen years in preservative, generally with a brown colouration and setae with a pale grey colour.

##### Etymology.

The specific epithet is a patronym for my father, Homero Giovanni Peñaherrera Zavala, who has always been a main pillar of my life, and always supported my crazy ideas like keeping aquariums and unconventional animals inside the house, which helped me grow my curiosity about nature.

##### Distribution.

*Cymbiapophysahomeroi* sp. nov. is only known from its type locality, on the Reserve Rio Guajalito, alongside the trail Los Españoles, Province of Santo Domingo de los Tsachilas, north of the western mountain range of the Andean Cordillera of Ecuador, at 2261 m.

##### Ecology.

The holotype of *Cymbiapophysahomeroi* sp. nov. was collected in the low montane evergreen forest of the Cordillera Occidental of the Andes of Ecuador, in the Northern Andes biogeographic province (Fig. 10).

##### Remarks.

The specimen is in a fragile state; thus, I did not measure scopulation, leg lengths, leg spination, and posterior lateral spinnerets segments to prevent fragmentation and loss of leg and pedipalp segments. Nevertheless, the most important characteristics to diagnose this new species are presented. The type specimen was found in the didactic collection of the ZSFQ and the original specimen label indicates “Guajalito. M. Costales’’. This specimen was collected by M. Costales during a student excursion to the Guajalito Reserve, managed by Vlastimil Zak, professor of Botany, in 2016 (Diego F. Cisneros-Heredia, in litt. 2023).

#### ﻿ Cymbiapophysa carmencita sp. nov.

Taxon classificationAnimaliaAraneaeTheraphosidae

D6CBBD29-CD20-5805-9432-9CDE0BC664BA

https://zoobank.org/C2549EAC-0BF7-4FB1-BA2E-2F5524CEC3A0

**Figure 5.**
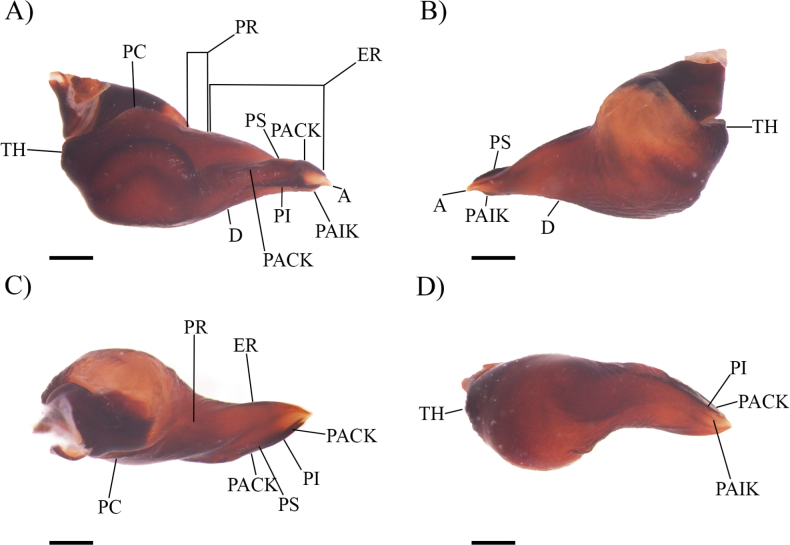
*Cymbiapophysacarmencita* sp. nov. male holotype (ZSFQ-i11578), palpal bulb (left hand side): **A** prolateral view **B** retrolateral view **C** dorsal view **D** ventral view. Scale bars: 0.5 mm.

**Figure 6.**
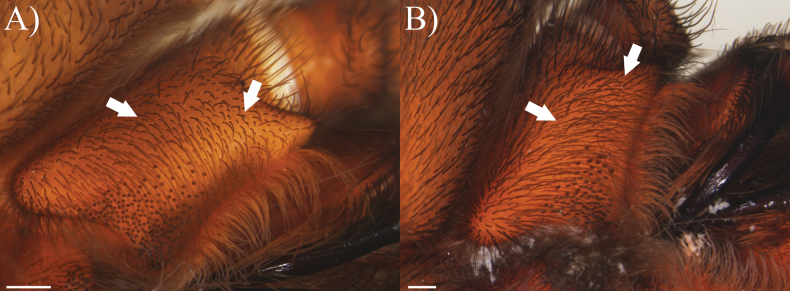
Maxillary spiniform setae (white arrow) **A***Cymbiapophysafalconi* sp. nov., male holotype (AE-0005) **B***Cymbiapophysacarmencita* sp. nov., male holotype (ZSFQ-i11578). Scale bars: 0.5 mm.

**Figure 7.**
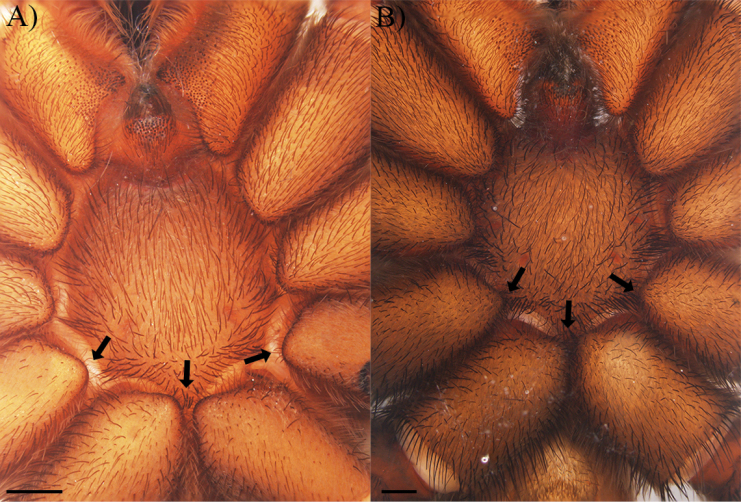
Prosoma showing ventro-basal dilatation of coxae (black arrows), ventral view **A***Cymbiapophysafalconi* sp. nov., male holotype (AE-0005) **B***Cymbiapophysacarmencita* sp. nov., male holotype (ZSFQ-i11578). Scale bars: 1 mm.

Figs 1 , 5 , 6B , 7B , 9A, B , 10

##### Examined material.

***Holotype***: Republic of Ecuador • 1 ♂; Province of Cotopaxi, Canton Pangua, Parish of El Corazón, Padrewasi; -1.2015, -78.9895, 2785 m a.s.l.; 25 February 2023; M. López-García, J. Montalvo, D. Brito-Zapata & C. Reyes-Puig leg.; ZFSQ-i11578.

##### Diagnosis.

*Cymbiapophysacarmencita* sp. nov. can be distinguished from other species by the morphology of the male palpal bulb from *C.falconi* sp. nov. by the presence a continuous and slightly serrated PACK keel, slightly serrated PI keel, developed A keel, PS as long as PI keel, absence of RI keel, and presence of Type III urticating setae (disjunct and distally serrated PACK keel, smooth PI keel, weakly developed A keel, PS keel longer than PI keel, well-developed RI keel, and absence of Type III urticating setae in *C.falconi* sp. nov.); *C.homeroi* sp. nov. by the slightly serrated PACK and PI keels, absence of RI keel, D weakly developed, and presence of Type III urticating setae (slightly serrated PACK keel and smooth PI keel, developed RS keel, weakly developed RI keel, D developed, and absence of Type III urticating setae in *C.homeroi*); from *C.velox* and *C.yimana* by the presence of a slightly serrated PACK and PI keels, PS keel as long as PI keel, and absence of RI keel (PACK keel(s) absent, smooth PI, PS keel as long as PI keel, and weakly developed RI in *C.velox* and *C.yimana* (Gabriel and Sherwood 2020; Sherwood et al. 2021a)); from *C.marimbai*, *C.magna*, and *C.seldeni* by the presence of a slightly serrated PACK keel, absence of a tibial apophysis, and D weakly developed (two PACK keels present, tibial apophysis present, and D well-developed in *C.marimbai*; PACK keel(s) absent, tibial apophysis present, and D developed in *C.magna*; PACK keel(s) absent, tibial apophysis present, and D weakly-developed in *C.seldeni* (Perafán and Valencia-Cuéllar 2018; Sherwood et al. 2021a; Sherwood and Gabriel 2023)). *Cymbiapophysacarmencita* sp. nov. can further be distinguished from all other species by the absence of a RS keel, developed A and PAIK keels (weakly developed RS and A keels, PAIK keel absent in *C.falconi* sp. nov., *C.magna*, *C.marimbai*, *C.velox*, and *C.yimana*, developed RS and A keels, PAIK keel absent in *C.homeroi* sp. nov.; weakly developed RS keel, well developed A keel, PAIK keel absent in *C.seldeni* (Perafán and Valencia-Cuéllar 2018; Gabriel and Sherwood 2020; Sherwood et al. 2021a; Sherwood and Gabriel 2023)).

##### Description.

***Male holotype*** (ZFSQ-i11578): Total length including chelicerae: 23.21. Carapace: length 10.38, width 8.59. Caput: raised. Ocular tubercle: slightly raised, length 0.98, width 1.46. Eyes: ALE > AME, AME > PLE, PLE > PME, anterior eye row slightly procurved, posterior row slightly recurved. Clypeus: narrow; clypeal fringe short. Fovea: straight. Chelicera: length 3.31, width 1.95. Abdomen: length 9.52, width 6.00. Maxilla with 112–129 cuspules covering approximately 40% of the proximal edge. Labium: length 1.76, width 0.95, with 37 cuspules most separated by 0.5–1.0× the width of a cuspule. Labio-sternal mounds joined along the entire base of the labium. Sternum: length 4.25, width 4.01, with three pairs of sigilla. Tarsi I–IV fully scopulate, tarsi I–II divided by narrow strip of longer and thicker setae, Tarsi III–IV divided by wide strip of longer and wider setae. Metatarsal scopulae: I 100%; II 70%; III 20%; IV 15%. Lengths of legs and palpal segments: see Table 2, legs IV, I, II, III. Spination: Leg I: femur p 0-0-2, v 1-1-1, d 1-2-1-2-1; patella p 0-2-0; tibia p 1-1-1-1-1, r 0-1-1-1-1-1, v 2-1-2-1-1-1 (3ap); metatarsus p 0-1-1-1, r 0-0-1, v 0-1-1-2 (4ap). Leg II: femur r 0-1-1-1-1, d 0-2-1-1-2; patella p 0-1-0, v 0-0-1; tibia p 1-0-1, r 1-1-1, v 2-1-2(3ap); metatarsus p 1-1-1, r 0-1-1-1, v 0-1-1-2-0(3ap). Leg III: femur r 0-1-1-1, d 2-1-2-1; patella p 0-1-0, r 0-1-0; tibia p 2-1-1-2, r 1-1-1, v 1-1-0(3ap), d 0-1-0; metatarsus p 1-1-1, r 0-1-1-1, v 0-1-1-1-1-1(3ap), d 0-0-2. Leg IV: femur p 0-1-1, d 1-1-1-1; patella p 0-1-0, r 0-1-0; tibia p 2-2-1-2-1, r 1-1-1, v 1-1-1-1(2ap), d 1-0-0; metatarsus p 1-1-1-1, r 0-1-1-1-1-1, v 1-1-1-1-1-1-1-1-1 (4ap). Pedipalp: femur p 0-0-1; patella p 0-1-0; tibia p 1-1-2-1-2, r 0-0-2. Palpal cymbium with rounded developed retrolateral apophysis (Fig. 1C). Leg I lacking tibial apophyses. Femur III laterally incrassate. Palpal tibia and metatarsus I straight. Posterior lateral spinnerets with three segments, basal 1.63, median 1.09, digitiform apical 2.60. Lateral median spinnerets with one segment. Palpal bulb (Fig. 5) with weakly-developed and quadrate TH. PS, PACK, and PI keels well-developed; A and PAIK developed. PS keel as long as PI keel. PACK keel extending less than a quarter of the embolus. PACK and PI keels slightly serrated. ER disjunct from PC forming a PR, PAR, RI, RS absent. PC narrow and constricted in posterior half. D weakly developed. Ventral face with rugulose area. Type Ib and III urticating setae present.

**Table 2.** *Cymbiapophysacarmencita* sp. nov. male holotype (ZFSQ-i11578), podomere measurements.

	Femur	Patella	Tibia	Metatarsus	Tarsus	Total
**I**	12.04	6.21	11.42	10.66	7.11	47.44
**II**	12.16	5.93	10.59	11.14	6.6	46.42
**III**	11.27	4.98	9.32	12.5	6.16	44.23
**IV**	13.37	5.21	12.38	16.89	7.48	55.33
**Palp**	6.82	3.17	6.96	-	2.4	19.35

##### Etymology.

The specific epithet *carmencita* is a noun in apposition and honours my mother, Carmen Beatriz Romero Palacios, and my sister, Carmen Emilia Peñaherrera-Romero. Although they have bad tempers, they have always supported and influenced me throughout my life.

##### Distribution.

*Cymbiapophysacarmencita* sp. nov. is only known from its type locality, near the sector of Padrewasi, Province of Cotopaxi, at 2785 m, in the central area of the Cordillera Occidental of the Andes of Ecuador.

##### Ecology.

The holotype of *Cymbiapophysacarmencita* sp. nov. was found under a log between a livestock intervention zone and *Guadua* patch in montane evergreen forest of the Cordillera Occidental of the Andes of Ecuador, in the Northern Andes biogeographic province (Fig. 10).

### ﻿Morphology observations

During the examination of the male specimens herein described as *C.falconi* sp. nov., *C.homeroi* sp. nov., and *C.carmencita* sp. nov. additional morphological characters that were not recorded for *Cymbiapophysa* were observed in relation to maxillae, coxae, trochanters, tibiae, and metatarsi segments of the three new species. Observations on these structures are presented in the following statements.

**Maxillae**: disperse thin maxillary spiniform setae present in the posterior margin and median to apical section (Fig. 6).

**Figure 8.**
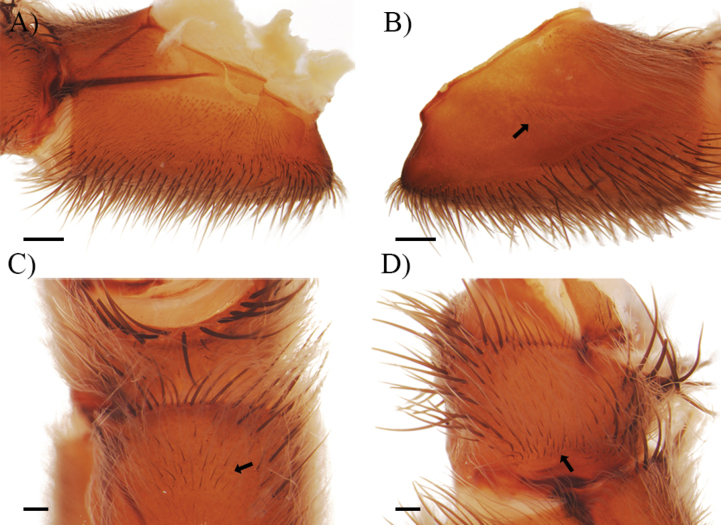
Coxa and trochanter I morphology and spiniform presence (black arrows) of *Cymbiapophysafalconi* sp. nov., male holotype (AE-0005) **A** coxa I showing coxal spinules, prolateral view **B** coxa I showing small group of short spiniform setae surrounded by elongated and more thinner setae, retrolateral view **C** coxa I showing dispersed group of short spiniform setae, dorsal view **D** trochanter I showing dispersed group of short spiniform setae, prolateral view. Scale bars: 0.5 mm (**A, B**); 0.2 mm (**C, D**).

**Coxae**: ventro-basal face of coxae generally dilatated (Fig. 7), coxae III and IV comparatively better to classify into different character states between species (e.g., developed ventro-basal dilatation of coxae III and IV in *C.falconi* sp. nov., weakly-developed ventro-basal dilatation of coxae III and IV in *C.carmencita*, [unknown state in *C.homeroi* sp. nov. due to the fragile state]). Different combination of modified setae were observed in coxae, including the presence of weakly-developed coxal spinules disposed at prolateral-ventral margin of coxae I and II (Fig. 8A), small group of short spiniform setae surrounded by elongated and more thinner setae disposed at retrolateral face of coxae I–III (Fig. 8B), and dispersed group of short spiniform setae disposed at dorsal face of coxae I–IV (Fig. 8C) resembling to those reported by Bertani et al. (2008) and Galleti-Lima and Guadanucci (2019).

**Trochanters**: dispersed group of short spiniform setae disposed at prolateral face of trochanter I–IV (Fig. 8D), tentatively resembling to those reported by Bertani et al. (2008) and Galleti-Lima and Guadanucci (2019).

**Figure 9.**
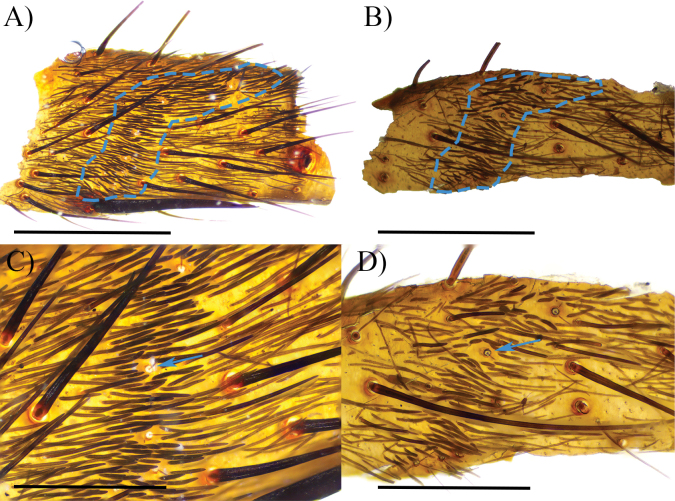
Group of short and coarse setae (light blue lines) and thickened trichobothria (light blue arrow) in metatarsus IV. *Cymbiapophysacarmencita* sp. nov. male holotype (ZSFQ-i11578) **A** retrolateral view of basal part **B** close up to structures. *Cymbiapophysafalconi* sp. nov. male paratype (AE-0004) **C** retrolateral view of basal part **D** close up to structures. Scale bars: 1.5 mm (**A, B**); 1 mm (**C, D**).

**Tibiae and metatarsi**: Tibiae I–IV and palpal tibial with small group of short and coarse setae with thickened trichobothria, extending in the basal part of prolateral and retrolateral faces (Fig. 9C, D). Metatarsi I–IV also with this group of setae and thickened trichobothria, extending from basal to medial from retrolateral face to dorsal face. Extension of this group of modified setae proportionally extends ~ 25% of the length of each segment in *C.falconi* sp. nov., *C.homeroi* sp. nov., and *C.carmencita* sp. nov.

### ﻿Key to species of *Cymbiapophysa* Gabriel & Sherwood, 2020 (males)

**Table:** 

1	Palpal bulb lacking PACK keel(s)	**2**
–	Palpal bulb with PACK keel(s)	**4**
2	Palpal bulb with PS keel longer than PI keel; PS, PI, and A keels developed; D developed	***C.magna* Sherwood, Gabriel, Brescovit & Lucas, 2021**
–	Palpal bulb with PS keel as long as PI keel; D weakly developed	**3**
3	Palpal bulb with PS and PI keels weakly developed	***C.velox* (Pocock, 1903)**
–	Palp bulb with PS and PI developed	**4**
4	Palpal bulb with A keel developed	***C.yimana* Gabriel & Sherwood, 2020**
–	Palpal bulb with A keel well-developed	***C.seldeni* Sherwood & Gabriel, 2023**
5	Palpal bulb with two PACK keels	***C.marimbai* (Perafán & Valencia-Cuéllar, 2018)**
–	Palpal bulb with only one PACK keel	**6**
6	Palpal bulb with disjunct PACK keel	**7**
–	Palpal bulb with continuous and slightly serrated PACK keel and smooth PI keel; RI keel well-developed and projected to prolateral face; D developed	***C.homeroi* sp. nov.**
7	Palpal bulb with distally and slightly serrated PACK keel and smooth PI keel; weakly developed A keel; PS keel longer than PI keel; PAIK absent	***C.falconi* sp. nov.**
–	Palpal bulb with slightly serrated PACK and PI keels; developed PAIK and A keels; PS keel as long as PI keel	***C.carmencita* sp. nov.**

## ﻿Discussion

The presence of maxillary spiniform setae and the group of short and coarse setae with thickened trichobothria on tibia and metatarsus I–IV could indicate additional morphological characters to differentiate *Cymbiapophysa* from other taxa that may present a similar palpal bulb morphology. Nevertheless, it would be advisable to evaluate the variation of these morphological characters in other species of *Cymbiapophysa* as also barb morphology with the use of Scanning electron microscope (SEM) images. The use of non-urticating setae morphology in Theraphosidae has already been used and currently appears reliable as a diagnostic character for some genera and species identification (Ferretti and Barneche 2013; Galleti-Lima and Guadanucci 2018, 2019; Kaderka et al. 2021; Ferretti et al. 2023). Currently in Andean taxa, maxillary spiniform setae are present in the following forms: (1) densely grouped in the posterior margin and median to apical section of the maxilla in *Bistriopelmamatuskai* Kaderka, 2015, *B.lamasi* Kaderka, 2015, and *B.titicaca* Kaderka, 2017, (2) grouped on distal half of maxilla in all species of *Antikuna* Kaderka, Ferretti, West, Lüddecke & Hüsser, 2021, *B.kiwicha* Nicoletta, Chaparro, Mamani, Ochoa, West & Ferretti, 2020, *B.peyoi* Nicoletta, Chaparro, Mamani, Ochoa, West & Ferretti, 2020, *Chinchaysuyuspinosa* Ferretti, Chaparro, Ochoa & West, 2023, and tentatively *B.fabianae* Quispe-Cocal & Kaderka, 2020, and (3) thin and dispersed in the posterior margin and median to apical section of maxilla in *C.falconi* sp. nov, *C.homeroi* sp. nov., *C.carmencita* sp. nov., and *Bumbapaunaka* Ferretti, 2021 (Kaderka 2015, 2017; Nicoletta et al. 2020; Quispe-Colca and Kaderka 2020; Ferretti 2021; Kaderka et al. 2021; Ferretti et al. 2023).

The discovery of small groups of spiniform setae in coxae I–III and groups of dispersed spiniform setae in trochanters of *Cymbiapophysa* could point out the existence of stridulatory setae and potentially complex communication behaviour. However, this came out the frame of this work, but it should be recommendable to realise long-term behaviour studies as also SEM images to accurately assess barbs morphology of each seta and correctly identify the type of modified setae.

Another example of modified setae found in the three new species of *Cymbiapophysa* are coxal spinules, another character used also for support genera diagnosis (e.g., *Bumba* Pérez-Miles, Bonaldo & Miglio, 2014). However, this character has only been treated as a double state character (presence or absence) instead of classifying in relation to the extension of coxae surface and type of modified spinule. Currently there is no standardised terminology for these structures, finding various terms in literature such as ‘’spike setae’’, ‘’spiniform setae’’, ‘’thorn-like’’, and ‘’stout setae’’ (Pérez-Miles 2000; Bertani and Carla-da-Silva 2003; Ferretti et al. 2011; Gallon and Wendt 2015; Hamilton et al. 2016). For this reason, and in relation to the use of the terms spiniform and stout setae for other modified setae, herein coxal spinules is proposed as a unified term with the following description: short spiniform setae with a notorious wider basal section almost pear-shaped and extremely short barbs (see Ferretti et al. 2011: fig. 10) restricted to the prolateral and retrolateral face of coxae; central and apical section of setae could be elongated but basal section become more thinner (e.g., weakly developed coxal spinules in *Cymbiapophysafalconi* sp. nov., and potentially in *Grammostoladoeringi* (Holmberg, 1881) (Fig. 8A; Ferretti et al. 2011: fig. 18) or central and apical section could be shorter or almost differentiable and basal section more wider (e.g., developed coxal spinules in *G.schulzei*, *Thrixopelma* spp., and *Aphonopelma* spp.; Ferretti et al. 2011; Hamilton et al. 2016; pers. obs.). The extension of coxal spinules in prolateral and retrolateral face and margins could also represent a potential state character for species and genera delimitation. Nevertheless, this should be tested with more specimens to confirm if there is any intra-specific variation and determine if there is any pattern that could function as supporting character for diagnosis.

**Figure 10.**
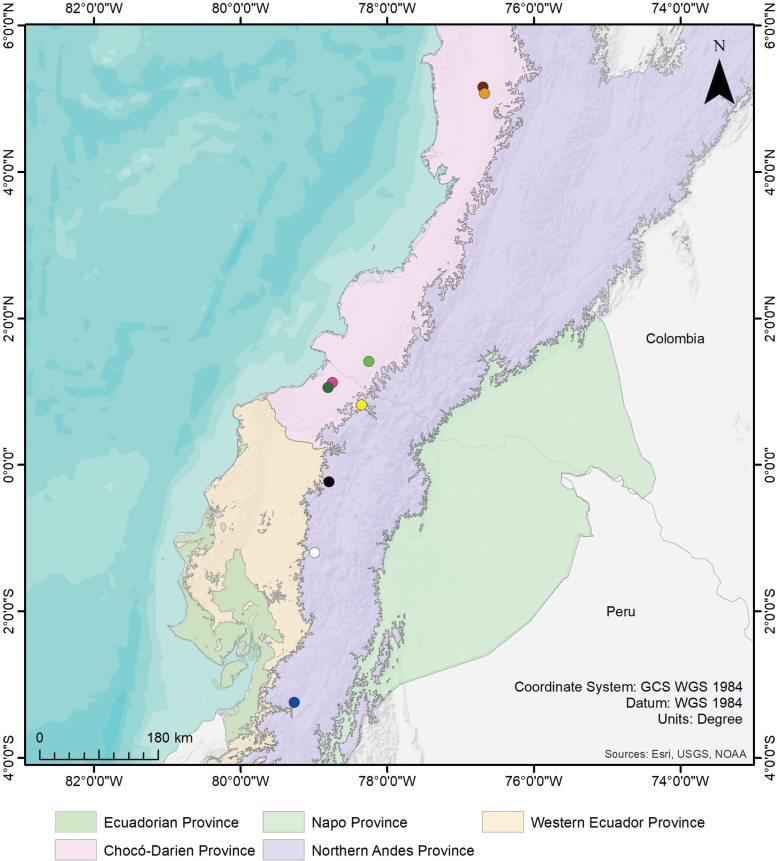
Distribution of the genus *Cymbiapophysa* Gabriel & Sherwood, 2020 including biogeographical regions of Ecuador and Colombia *sensu*Morrone 2014 and Cisneros-Heredia 2019. Blue circle = Valley of Yunguilla, type locality of *C.falconi* sp. nov.; Black circle = Reserve Rio Guajalito, type locality of *C.homeroi* sp. nov.; White circle = Padrewasi, type locality of *C.carmencita* sp. nov.; Yellow circle = Paramba, type locality of *C.velox*; Pink circle = Pambelar, historic record of *C.velox*; Dark green circle = Carondelet, type locality of *C.seldeni*; Light green circle = Reserva Natural Biotopo Selva Húmeda, type locality of *C.marimbai*; Dark brown circle = Itsmina, possible occurrence of *C.magna*; Light brown circle = Andagoya, possible occurrence of *C.magna*.

Until now the ventro-basal dilatation of coxae was recorded only for the genus *Hemirragus* Simon, 1903, the discovery of this character in *Cymbiapophysa* could potentially represent another diagnostic character at genus and species level with the combination of palpal bulb morphology (Mendoza Marroquín 2014). As previously mentioned, ventro-basal dilatation of coxae shows different character states between *C.falconi* sp. nov. and *C.carmencita* sp. nov., pointing out the potential use of this character as another supplementary character for species delimitation. Undoubtedly additional morphologic characters could help provide more information in case of conflicting or not well-understood Andean theraphosid spiders.

The production of taxonomic knowledge about tarantulas from the northern Andes has advanced enormously, providing redescriptions, new species, and new genera (e.g., Perafán et al. 2015, 2016; Gabriel and Sherwood 2020; Sherwood et al. 2021b; Cifuentes and Bertani 2022; Sherwood and Gabriel 2022; Echeverri et al. 2023). However, there are still clarifications needed for the known localities for some species, often due to the limited information on historical specimens. This occurs in the case *C.magna* where the information on the type locality is restricted to a general area along the Río San Juan (see below). The lack of this knowledge impedes the study of this species by future researchers. Additional information on the known localities of each species will be presented in the following paragraph.

*Cymbiapophysamagna* Sherwood, Gabriel, Brescovit & Lucas, 2021 was recently described by Sherwood et al. (2021a) based on a holotype and paratype male collected by Emilio J. Pampana in Colombia, somewhere in the central Chocó, Río San Juan, alongside the mining camps of Compania Minera Chocó Pacífico. Following the same line of thought of Sherwood et al. (2021a), it is most likely that Pampana only passed between the camps of this mining company. Fortunately, the company only had two camps around the San Juan River: Itsmina and Andagoya (Rada 1934; Leal León 2009). After reviewing gazetteers available in SiB Colombia (2020) of herpetology collections, both localities can be restricted as follows: (1) Itsmina (5.1600, -76.6927), department of Chocó, 155 m elevation, foothill humid gallery forest of the Chocó-Darien Province. (2) Andagoya (5.0722, -76.6717), 50 m elevation, foothill humid gallery forest of the Chocó-Darien Province. It is possible that the holotype of *C.magna* was collected at one of these two localities or somewhere in between, tentatively along the main transport routes. As Sherwood et al. (2021a) stated, with this additional information, future researchers should examine more specimens to confirm the type locality and hopefully present the description of the female.

*Cymbiapophysa* seems to be widely spread in two biogeographical provinces of the western mountain range of the Andean cordillera of Ecuador and the Western Cordillera of Colombia (Fig. 10), with an altitudinal range of 12–2785 m; being *C.carmencita* sp. nov. with the highest record for the genus. The species *C.magna* and *C.marimbai* (Perafán & Valencia-Cuéllar, 2018) are distributed in the Chocó-Darien Province in central and southern Colombia, respectively. The species *C.velox* and *C.seldeni* are distributed in the Chocó-Darien Province in northern Ecuador. The species *C.falconi* sp. nov. is distributed in the Western Ecuador Province in southern Ecuador, meanwhile *C.homeroi* sp. nov. and *C.carmencita* sp. nov. are distributed in the Northern Andes Province in central and southern Ecuador, respectively.

Although so little is known about the distribution of each species of *Cymbiapophysa*, it is very likely that across western Ecuador and Colombia there is great potential for new species of *Cymbiapophysa* to be found by future researchers across these three biogeographical provinces. Furthermore, the possibility of sympatric (e.g., *Pamphobeteusvespertinus* and *P.augusti*, Sherwood et al. 2022) and parapatric species (e.g., *C.falconi* sp. nov. and potential presence of additional new species of *Cymbiapophysa* inside the Jubones river basin and sidestreams) of theraphosids of the same genus must be considered between altitudinal gradients. Meaning that it is suggestable to carry out extensive fieldwork in order to assess a better understanding on the diversity of this neotropical clade and even the use of integrative methodologies for species delimitation.

## Supplementary Material

XML Treatment for Cymbiapophysa falconi

XML Treatment for Cymbiapophysa homeroi

XML Treatment for Cymbiapophysa carmencita

## ﻿Additional information

### Author ORCIDs

Pedro Peñaherrera-R. https://orcid.org/0000-0001-9285-3403

### Data availability

All of the data that support the findings of this study are available in the main text.
